# 2-(1*H*-Benzimidazol-2-yl)-*N*-[(*E*)-(dimethyl­amino)­methyl­idene]benzene­sulfonamide

**DOI:** 10.1107/S1600536812025159

**Published:** 2012-06-13

**Authors:** Adnan Ashraf, M. Nawaz Tahir, Waseeq Ahmad Siddiqui, Nadia Perveen

**Affiliations:** a University of Sargodha, Department of Chemistry, Sargodha, Pakistan; b University of Sargodha, Department of Physics, Sargodha, Pakistan

## Abstract

The asymmetric unit of the title compound, C_16_H_16_N_4_O_2_S, contains two mol­ecules (*A* and *B*) with similar conformations: the benzene rings are oriented at dihedral angles of 80.94 (10)° and 84.54 (10)° with their adjacent 1*H*-benzimidazole groups. In the crystal, the mol­ecules are connected by N—H⋯N hydrogen bonds, forming separate *C*(4) chains of both the *A* and *B* mol­ecules along [010]. The *A* and *B* chains are cross-linked by several C—H⋯O inter­actions involving the benzene rings and the sulfonyl groups, which lead to *R*_2_^1^(5) loops in the linkage of the chains.

## Related literature

For a related structure, see: Esparza-Ruiz *et al.* (2010 ▶).
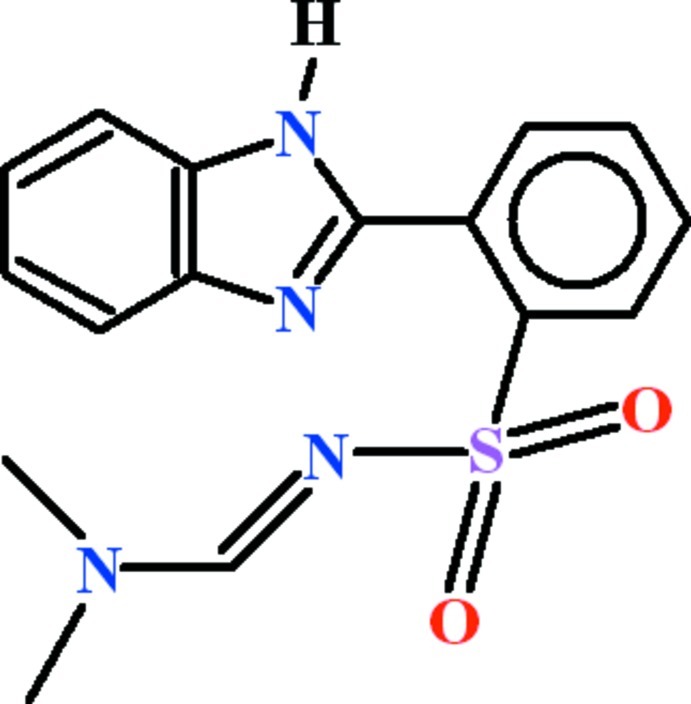


## Experimental

### 

#### Crystal data

C_16_H_16_N_4_O_2_S
*M* *_r_* = 328.39
Monoclinic, 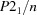

*a* = 15.630 (5) Å
*b* = 10.003 (4) Å
*c* = 22.122 (5) Å
β = 110.657 (5)°
*V* = 3236.3 (18) Å^3^
*Z* = 8
Mo *K*α radiation
μ = 0.22 mm^−1^
*T* = 296 K
0.28 × 0.20 × 0.18 mm

#### Data collection

Bruker Kappa APEXII CCD diffractometer
Absorption correction: multi-scan (*SADABS*; Bruker, 2005 ▶) *T*_min_ = 0.930, *T*_max_ = 0.952
26702 measured reflections
6347 independent reflections
3328 reflections with *I* > 2σ(*I*)
*R*_int_ = 0.070

#### Refinement

*R*[*F* ^2^ > 2σ(*F* ^2^)] = 0.059
*wR*(*F* ^2^) = 0.159
*S* = 1.01
6347 reflections
419 parameters
H-atom parameters constrained
Δρ_max_ = 0.34 e Å^−3^
Δρ_min_ = −0.32 e Å^−3^

### 

Data collection: *APEX2* (Bruker, 2009 ▶); cell refinement: *SAINT* (Bruker, 2009 ▶); data reduction: *SAINT*; program(s) used to solve structure: *SHELXS97* (Sheldrick, 2008 ▶); program(s) used to refine structure: *SHELXL97* (Sheldrick, 2008 ▶); molecular graphics: *ORTEP-3 for Windows* (Farrugia, 1997 ▶) and *PLATON* (Spek, 2009 ▶); software used to prepare material for publication: *WinGX* (Farrugia, 1999 ▶) and *PLATON*.

## Supplementary Material

Crystal structure: contains datablock(s) global, I. DOI: 10.1107/S1600536812025159/hb6832sup1.cif

Structure factors: contains datablock(s) I. DOI: 10.1107/S1600536812025159/hb6832Isup2.hkl

Supplementary material file. DOI: 10.1107/S1600536812025159/hb6832Isup3.cml

Additional supplementary materials:  crystallographic information; 3D view; checkCIF report

## Figures and Tables

**Table 1:** Hydrogen-bond geometry (Å, °)

*D*—H⋯*A*	*D*—H	H⋯*A*	*D*⋯*A*	*D*—H⋯*A*
N1—H1⋯N2^i^	0.86	2.11	2.964 (3)	174
N5—H5*A*⋯N6^ii^	0.86	2.10	2.955 (3)	178
C9—H9⋯O4^i^	0.93	2.56	3.153 (4)	122
C10—H10⋯O4^i^	0.93	2.57	3.153 (4)	121
C25—H25⋯O2^ii^	0.93	2.47	3.114 (5)	127

Symmetry codes: (i) 
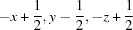
; (ii) 
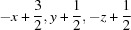
.

**Fig. 1.**
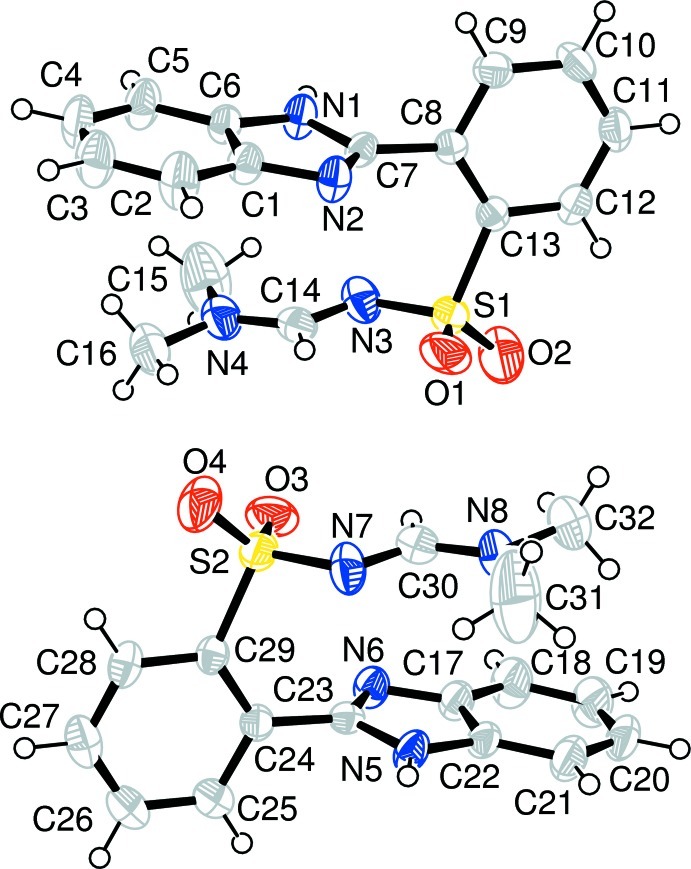
View of the title compound with displecement ellipsoids drawn at the 50% probability level.

**Fig. 2.**
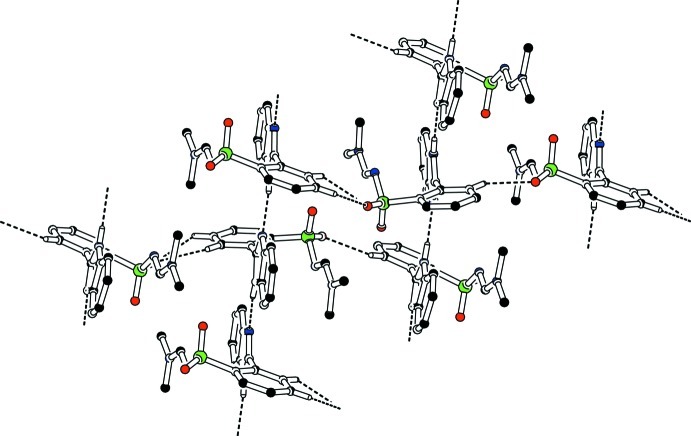
The partial packing (PLATON; Spek, 2009) which shows that molecules form C(4) chains and are interlinked. The H-atoms not involved in H-bondings are omitted for clarity.

## supplementary crystallographic information

### Comment

The title compound (I), (Fig. 1) has been prepared in an attempt to attach benzenesulfonyl chloride with 2-(1*H*-benzimidazol-2-yl) benzenesulfonamide (Crystal structure has been determined) in the dimethylformamide.

The crystal structures of 2-(1*H*-benzimidazol-3-ium-2-yl)benzenesulfonate dimethylsulfoxide solvate (Esparza-Ruiz *et al.*, 2010) has been published which is related to (I) upto some extent.

In (I), two molecules (M1 and M2) in the asymmetric unit are present, which differ slightly from each other geometrically. In molecule M1, the group A (C1—C7/N1/N2) of 1*H*-benzimidazole, benzene ring B (C8—C13) and group C (N3/C14/N4/C15/C16) of *N*,*N*-dimethylimidoformamide moiety are planar with r. m. s. deviation of 0.0108 Å, 0.0046 Å and 0.0093 Å, respectively. The dihedral angle between A/B, A/C and B/C is 80.94 (10)°, 12.34 (4)° and 83.76 (18)°, respectively. The sulfonyl group D (O1/S1/O2) is of course planar. The dihedral angle between B/D and C/D is 70.86 (14)° and 53.88 (13)°, respectively. In second molecule M2, the similar groups E (C17—C23/N5/N6), F (C24—C29) and G (N7/C30/N8/C31/C32) are also planar with r. m. s. deviation of 0.0160 Å, 0.0054 Å and 0.0122 Å, respectively. The dihedral angle between E/F, E/G and F/G is 84.54 (10)°, 12.68 (8)° and 83.22 (20)°, respectively. In M2, dihedral angle between F/H and G/H is 69.47 (14)° and 54.53 (13)°, respectively where H (O3/S2/O4) is the sulfonyl group. Both molecules are interlinked with themselves with C (4) chains due to classical H–bonding of N—H···N type (Table 1, Fig. 2). These infinte one-dimensional chains exist along [010]. The polymeric chains are interlinked with each other through benzene ring and the sulfonyl groups due to H–bonding of C—H···O type in a different manner. There exist *R*_2_^1^(5) ring motif in the linkage of polymeric chains.

### Experimental

The 2-[*o*-(sulfamoyl)phenyl]benzimidazole (0.1 g, 0.37 mmol) in dimethylformamide (2 ml) was disolved to get a clear solution. Benzenesulfonyl chloride (0.065 g, 0.37 mmol) was added with catalytic amount of potassium carbonate to this solution and subjected to reflux for 2 h. The resulting solution was quenched in ice-cold distilled water (100 ml). Extracted the aqueous layer with ethyl acetate (3 × 25 ml) and dried the organic layer over anhydrous sodium sulfate to get light brown powder (0.11 g, 90.5%). The powder was recrystallized in methanol to get the light brown prisms of (I).

m.p. 593–594 K.

### Refinement

The H-atoms were positioned geometrically at C—H = 0.93—0.96, nd N—H = 0.86 Å, respectively and included in the refinement as riding with *U*_iso_(H) = *xU*_eq_(C, N), where *x* = 1.5 for metyl H-atoms and *x* = 1.2 for all other H-atoms.

### Crystal data

**Table:**

C_16_H_16_N_4_O_2_S	*F*(000) = 1376
*M**_r_* = 328.39	*D*_x_ = 1.348 Mg m^−^^3^
Monoclinic, *P*2_1_/*n*	Mo *K*α radiation, λ = 0.71073 Å
Hall symbol: -P 2yn	Cell parameters from 3328 reflections
*a* = 15.630 (5) Å	θ = 2.0–26.0°
*b* = 10.003 (4) Å	µ = 0.22 mm^−^^1^
*c* = 22.122 (5) Å	*T* = 296 K
β = 110.657 (5)°	Prism, light brown
*V* = 3236.3 (18) Å^3^	0.28 × 0.20 × 0.18 mm
*Z* = 8	

### Data collection

**Table:**

Bruker Kappa APEXII CCD diffractometer	6347 independent reflections
Radiation source: fine-focus sealed tube	3328 reflections with *I* > 2σ(*I*)
Graphite monochromator	*R*_int_ = 0.070
Detector resolution: 8.00 pixels mm^-1^	θ_max_ = 26.0°, θ_min_ = 2.0°
ω scans	*h* = −19→19
Absorption correction: multi-scan (*SADABS*; Bruker, 2005)	*k* = −12→12
*T*_min_ = 0.930, *T*_max_ = 0.952	*l* = −27→22
26702 measured reflections	

### Refinement

**Table:**

Refinement on *F*^2^	Primary atom site location: structure-invariant direct methods
Least-squares matrix: full	Secondary atom site location: difference Fourier map
*R*[*F*^2^ > 2σ(*F*^2^)] = 0.059	Hydrogen site location: inferred from neighbouring sites
*wR*(*F*^2^) = 0.159	H-atom parameters constrained
*S* = 1.01	*w* = 1/[σ^2^(*F*_o_^2^) + (0.0638*P*)^2^ + 0.4292*P*] where *P* = (*F*_o_^2^ + 2*F*_c_^2^)/3
6347 reflections	(Δ/σ)_max_ < 0.001
419 parameters	Δρ_max_ = 0.34 e Å^−^^3^
0 restraints	Δρ_min_ = −0.32 e Å^−^^3^

### Special details

**Table:**

Geometry. Bond distances, angles *etc*. have been calculated using the rounded fractional coordinates. All su's are estimated from the variances of the (full) variance-covariance matrix. The cell e.s.d.'s are taken into account in the estimation of distances, angles and torsion angles
Refinement. Refinement of *F*^2^ against ALL reflections. The weighted *R*-factor *wR* and goodness of fit *S* are based on *F*^2^, conventional *R*-factors *R* are based on *F*, with *F* set to zero for negative *F*^2^. The threshold expression of *F*^2^ > σ(*F*^2^) is used only for calculating *R*-factors(gt) *etc*. and is not relevant to the choice of reflections for refinement. *R*-factors based on *F*^2^ are statistically about twice as large as those based on *F*, and *R*- factors based on ALL data will be even larger.

### Fractional atomic coordinates and isotropic or equivalent isotropic displacement parameters (Å^2^)

**Table:**

	*x*	*y*	*z*	*U*_iso_*/*U*_eq_	
S1	0.37734 (6)	0.20738 (11)	0.16157 (4)	0.0540 (4)	
O1	0.38444 (16)	0.3454 (2)	0.18274 (13)	0.0766 (11)	
O2	0.41107 (17)	0.1781 (4)	0.11124 (13)	0.1060 (16)	
N1	0.25436 (18)	0.0481 (2)	0.27589 (11)	0.0378 (9)	
N2	0.25592 (18)	0.2709 (2)	0.27357 (12)	0.0369 (9)	
N3	0.42077 (18)	0.1030 (3)	0.21989 (13)	0.0507 (10)	
N4	0.49756 (19)	0.0855 (3)	0.32963 (14)	0.0548 (11)	
C1	0.2819 (2)	0.2276 (3)	0.33732 (15)	0.0362 (11)	
C2	0.3058 (3)	0.2996 (3)	0.39450 (15)	0.0529 (14)	
C3	0.3299 (3)	0.2312 (4)	0.45146 (16)	0.0563 (14)	
C4	0.3314 (3)	0.0922 (3)	0.45294 (15)	0.0541 (16)	
C5	0.3069 (3)	0.0181 (3)	0.39697 (15)	0.0510 (14)	
C6	0.2813 (2)	0.0883 (3)	0.33941 (14)	0.0341 (11)	
C7	0.24058 (19)	0.1606 (3)	0.23952 (14)	0.0293 (10)	
C8	0.20535 (19)	0.1538 (3)	0.16793 (13)	0.0278 (9)	
C9	0.1123 (2)	0.1313 (3)	0.13747 (15)	0.0398 (11)	
C10	0.0741 (2)	0.1237 (3)	0.07082 (16)	0.0479 (11)	
C11	0.1275 (2)	0.1359 (3)	0.03388 (15)	0.0429 (11)	
C12	0.2199 (2)	0.1569 (3)	0.06293 (15)	0.0388 (11)	
C13	0.25898 (19)	0.1670 (3)	0.12983 (14)	0.0304 (10)	
C14	0.4588 (2)	0.1557 (3)	0.27730 (16)	0.0472 (11)	
C15	0.4997 (3)	−0.0594 (4)	0.3281 (2)	0.1017 (19)	
C16	0.5413 (3)	0.1492 (4)	0.39179 (17)	0.0774 (16)	
S2	0.62238 (5)	0.50313 (10)	0.33544 (4)	0.0466 (3)	
O3	0.62214 (15)	0.3695 (2)	0.31038 (12)	0.0628 (9)	
O4	0.58453 (16)	0.5169 (3)	0.38516 (12)	0.0784 (13)	
N5	0.73966 (18)	0.6823 (2)	0.22561 (11)	0.0399 (9)	
N6	0.75783 (18)	0.4613 (2)	0.22988 (12)	0.0400 (9)	
N7	0.57612 (18)	0.6125 (3)	0.28025 (13)	0.0495 (10)	
N8	0.5008 (2)	0.6440 (4)	0.17119 (14)	0.0673 (13)	
C17	0.7302 (2)	0.5032 (3)	0.16597 (15)	0.0412 (11)	
C18	0.7158 (3)	0.4298 (4)	0.10993 (16)	0.0602 (14)	
C19	0.6876 (3)	0.4976 (4)	0.05251 (17)	0.0652 (16)	
C20	0.6720 (3)	0.6343 (4)	0.04889 (16)	0.0612 (16)	
C21	0.6871 (3)	0.7089 (3)	0.10395 (16)	0.0539 (14)	
C22	0.7180 (2)	0.6412 (3)	0.16255 (14)	0.0382 (11)	
C23	0.76194 (19)	0.5718 (3)	0.26304 (14)	0.0309 (10)	
C24	0.7940 (2)	0.5793 (3)	0.33451 (14)	0.0326 (10)	
C25	0.8858 (2)	0.6104 (3)	0.36720 (16)	0.0478 (11)	
C26	0.9208 (2)	0.6157 (4)	0.43360 (17)	0.0591 (14)	
C27	0.8658 (3)	0.5928 (3)	0.46862 (17)	0.0539 (12)	
C28	0.7741 (2)	0.5633 (3)	0.43755 (15)	0.0415 (11)	
C29	0.7384 (2)	0.5552 (3)	0.37065 (14)	0.0320 (10)	
C30	0.5412 (2)	0.5667 (4)	0.22150 (17)	0.0555 (16)	
C31	0.4949 (4)	0.7876 (5)	0.1769 (2)	0.122 (3)	
C32	0.4600 (3)	0.5844 (5)	0.10749 (19)	0.108 (2)	
H1	0.24759	−0.03292	0.26196	0.0453*	
H2	0.30546	0.39255	0.39403	0.0639*	
H3	0.34568	0.27844	0.49005	0.0673*	
H4	0.34927	0.04845	0.49255	0.0651*	
H5	0.30761	−0.07483	0.39780	0.0608*	
H9	0.07525	0.12129	0.16212	0.0479*	
H10	0.01148	0.11022	0.05105	0.0573*	
H11	0.10149	0.12994	−0.01087	0.0516*	
H12	0.25644	0.16446	0.03780	0.0467*	
H14	0.45815	0.24824	0.28106	0.0564*	
H15A	0.45358	−0.09097	0.28921	0.1522*	
H15B	0.48820	−0.09457	0.36489	0.1522*	
H15C	0.55872	−0.08863	0.32916	0.1522*	
H16A	0.53553	0.24449	0.38678	0.1162*	
H16B	0.60491	0.12542	0.40845	0.1162*	
H16C	0.51255	0.11996	0.42130	0.1162*	
H5A	0.73910	0.76310	0.23871	0.0478*	
H18	0.72508	0.33784	0.11139	0.0719*	
H19	0.67852	0.45024	0.01457	0.0784*	
H20	0.65107	0.67604	0.00876	0.0736*	
H21	0.67706	0.80070	0.10202	0.0649*	
H25	0.92372	0.62779	0.34384	0.0575*	
H26	0.98240	0.63487	0.45476	0.0711*	
H27	0.88990	0.59712	0.51348	0.0645*	
H28	0.73658	0.54898	0.46149	0.0496*	
H30	0.54482	0.47545	0.21474	0.0667*	
H31A	0.53722	0.81604	0.21800	0.1837*	
H31B	0.43392	0.81161	0.17345	0.1837*	
H31C	0.50972	0.83024	0.14296	0.1837*	
H32A	0.48518	0.62559	0.07828	0.1617*	
H32B	0.39504	0.59798	0.09207	0.1617*	
H32C	0.47291	0.49031	0.11006	0.1617*	

### Atomic displacement parameters (Å^2^)

**Table:**

	*U*^11^	*U*^22^	*U*^33^	*U*^12^	*U*^13^	*U*^23^
S1	0.0310 (5)	0.0841 (8)	0.0447 (6)	−0.0017 (5)	0.0108 (4)	0.0174 (5)
O1	0.0589 (17)	0.0562 (18)	0.087 (2)	−0.0297 (13)	−0.0085 (15)	0.0247 (14)
O2	0.0421 (16)	0.230 (4)	0.0547 (19)	0.022 (2)	0.0280 (14)	0.026 (2)
N1	0.0621 (18)	0.0204 (13)	0.0280 (15)	−0.0069 (12)	0.0123 (13)	−0.0025 (11)
N2	0.0582 (18)	0.0215 (14)	0.0280 (15)	−0.0024 (12)	0.0116 (13)	−0.0009 (11)
N3	0.0426 (16)	0.0581 (19)	0.0441 (19)	0.0099 (14)	0.0063 (14)	0.0020 (15)
N4	0.0478 (17)	0.058 (2)	0.0480 (19)	−0.0014 (15)	0.0037 (15)	0.0097 (16)
C1	0.0462 (19)	0.0310 (18)	0.0311 (19)	−0.0014 (14)	0.0133 (15)	−0.0018 (14)
C2	0.086 (3)	0.0307 (19)	0.036 (2)	−0.0013 (19)	0.014 (2)	−0.0063 (16)
C3	0.082 (3)	0.052 (2)	0.033 (2)	−0.001 (2)	0.018 (2)	−0.0100 (17)
C4	0.074 (3)	0.056 (3)	0.028 (2)	−0.002 (2)	0.0125 (18)	0.0081 (17)
C5	0.081 (3)	0.0332 (19)	0.035 (2)	−0.0088 (18)	0.0156 (19)	0.0047 (16)
C6	0.0458 (19)	0.0259 (18)	0.0288 (18)	−0.0067 (14)	0.0109 (15)	−0.0004 (14)
C7	0.0359 (17)	0.0244 (16)	0.0289 (17)	−0.0020 (14)	0.0132 (14)	0.0021 (14)
C8	0.0324 (16)	0.0200 (16)	0.0293 (17)	−0.0010 (13)	0.0089 (14)	−0.0007 (12)
C9	0.0370 (18)	0.045 (2)	0.039 (2)	−0.0078 (15)	0.0154 (16)	−0.0052 (15)
C10	0.0317 (18)	0.058 (2)	0.041 (2)	−0.0067 (16)	−0.0034 (16)	−0.0018 (17)
C11	0.050 (2)	0.043 (2)	0.0280 (19)	−0.0027 (16)	0.0042 (17)	0.0017 (15)
C12	0.049 (2)	0.0368 (19)	0.0322 (19)	−0.0013 (16)	0.0162 (16)	0.0034 (14)
C13	0.0319 (16)	0.0262 (16)	0.0317 (18)	0.0009 (13)	0.0095 (14)	0.0019 (13)
C14	0.0338 (18)	0.053 (2)	0.050 (2)	−0.0061 (16)	0.0089 (17)	0.0087 (18)
C15	0.126 (4)	0.066 (3)	0.082 (3)	0.028 (3)	−0.002 (3)	0.010 (2)
C16	0.077 (3)	0.087 (3)	0.046 (2)	−0.024 (2)	−0.006 (2)	0.009 (2)
S2	0.0311 (4)	0.0672 (7)	0.0420 (5)	0.0038 (4)	0.0134 (4)	0.0173 (5)
O3	0.0529 (15)	0.0439 (15)	0.0805 (19)	−0.0146 (12)	0.0099 (14)	0.0084 (13)
O4	0.0459 (15)	0.146 (3)	0.0526 (17)	0.0202 (16)	0.0291 (14)	0.0309 (16)
N5	0.0627 (18)	0.0266 (14)	0.0311 (15)	−0.0061 (13)	0.0176 (13)	0.0016 (12)
N6	0.0562 (18)	0.0334 (15)	0.0306 (15)	−0.0038 (13)	0.0155 (13)	−0.0043 (12)
N7	0.0475 (17)	0.0610 (19)	0.0343 (17)	0.0147 (14)	0.0072 (14)	0.0086 (14)
N8	0.0533 (19)	0.099 (3)	0.0369 (19)	−0.0115 (19)	0.0002 (15)	0.0157 (18)
C17	0.057 (2)	0.0347 (19)	0.0308 (19)	−0.0103 (16)	0.0141 (16)	−0.0033 (15)
C18	0.091 (3)	0.047 (2)	0.040 (2)	−0.010 (2)	0.020 (2)	−0.0105 (18)
C19	0.092 (3)	0.068 (3)	0.033 (2)	−0.021 (2)	0.019 (2)	−0.015 (2)
C20	0.082 (3)	0.070 (3)	0.028 (2)	−0.025 (2)	0.015 (2)	0.0067 (18)
C21	0.075 (3)	0.046 (2)	0.037 (2)	−0.0132 (19)	0.015 (2)	0.0075 (17)
C22	0.051 (2)	0.037 (2)	0.0264 (18)	−0.0136 (15)	0.0134 (15)	−0.0023 (14)
C23	0.0309 (16)	0.0298 (17)	0.0320 (18)	−0.0047 (14)	0.0112 (14)	−0.0010 (14)
C24	0.0362 (17)	0.0317 (18)	0.0284 (18)	−0.0015 (14)	0.0097 (14)	0.0006 (14)
C25	0.0394 (19)	0.059 (2)	0.042 (2)	−0.0086 (17)	0.0107 (17)	−0.0008 (17)
C26	0.041 (2)	0.079 (3)	0.046 (2)	−0.0135 (19)	0.0015 (19)	−0.003 (2)
C27	0.059 (2)	0.059 (2)	0.033 (2)	0.002 (2)	0.0029 (19)	−0.0040 (17)
C28	0.052 (2)	0.047 (2)	0.0290 (19)	0.0068 (16)	0.0185 (16)	0.0038 (15)
C29	0.0338 (17)	0.0313 (17)	0.0294 (18)	0.0046 (13)	0.0093 (14)	0.0053 (13)
C30	0.038 (2)	0.073 (3)	0.050 (3)	−0.0114 (18)	0.0088 (18)	0.012 (2)
C31	0.155 (6)	0.104 (4)	0.078 (4)	0.053 (4)	0.004 (4)	0.029 (3)
C32	0.093 (4)	0.164 (5)	0.043 (3)	−0.056 (3)	−0.005 (2)	0.011 (3)

### Geometric parameters (Å, º)

**Table:**

S1—O1	1.450 (2)	C4—H4	0.9300
S1—O2	1.420 (3)	C5—H5	0.9300
S1—N3	1.612 (3)	C9—H9	0.9300
S1—C13	1.778 (3)	C10—H10	0.9300
S2—O4	1.428 (3)	C11—H11	0.9300
S2—N7	1.609 (3)	C12—H12	0.9300
S2—C29	1.780 (3)	C14—H14	0.9300
S2—O3	1.447 (2)	C15—H15C	0.9600
N1—C6	1.377 (4)	C15—H15A	0.9600
N1—C7	1.356 (4)	C15—H15B	0.9600
N2—C7	1.310 (4)	C16—H16C	0.9600
N2—C1	1.392 (4)	C16—H16B	0.9600
N3—C14	1.308 (4)	C16—H16A	0.9600
N4—C14	1.306 (4)	C17—C22	1.392 (4)
N4—C16	1.450 (5)	C17—C18	1.389 (5)
N4—C15	1.451 (5)	C18—C19	1.369 (5)
N1—H1	0.8600	C19—C20	1.386 (6)
N5—C22	1.377 (4)	C20—C21	1.376 (5)
N5—C23	1.351 (4)	C21—C22	1.390 (4)
N6—C17	1.389 (4)	C23—C24	1.482 (4)
N6—C23	1.316 (4)	C24—C29	1.394 (5)
N7—C30	1.303 (5)	C24—C25	1.395 (5)
N8—C32	1.454 (5)	C25—C26	1.376 (5)
N8—C30	1.319 (5)	C26—C27	1.364 (6)
N8—C31	1.448 (6)	C27—C28	1.385 (6)
N5—H5A	0.8600	C28—C29	1.388 (4)
C1—C2	1.387 (4)	C18—H18	0.9300
C1—C6	1.394 (4)	C19—H19	0.9300
C2—C3	1.365 (5)	C20—H20	0.9300
C3—C4	1.391 (5)	C21—H21	0.9300
C4—C5	1.376 (4)	C25—H25	0.9300
C5—C6	1.384 (4)	C26—H26	0.9300
C7—C8	1.484 (4)	C27—H27	0.9300
C8—C9	1.389 (5)	C28—H28	0.9300
C8—C13	1.389 (4)	C30—H30	0.9300
C9—C10	1.384 (5)	C31—H31A	0.9600
C10—C11	1.364 (5)	C31—H31B	0.9600
C11—C12	1.374 (5)	C31—H31C	0.9600
C12—C13	1.391 (4)	C32—H32A	0.9600
C2—H2	0.9300	C32—H32B	0.9600
C3—H3	0.9300	C32—H32C	0.9600
			
O1—S1—O2	116.3 (2)	N3—C14—H14	118.00
O1—S1—N3	113.32 (16)	N4—C14—H14	118.00
O1—S1—C13	107.12 (16)	N4—C15—H15B	109.00
O2—S1—N3	109.02 (19)	N4—C15—H15A	109.00
O2—S1—C13	105.41 (16)	N4—C15—H15C	109.00
N3—S1—C13	104.75 (15)	H15B—C15—H15C	109.00
O3—S2—N7	113.45 (15)	H15A—C15—H15C	109.00
O3—S2—C29	107.61 (15)	H15A—C15—H15B	109.00
O4—S2—N7	108.77 (16)	H16A—C16—H16B	109.00
O4—S2—C29	105.65 (15)	H16B—C16—H16C	109.00
N7—S2—C29	104.41 (15)	N4—C16—H16B	109.00
O3—S2—O4	116.02 (17)	H16A—C16—H16C	109.00
C6—N1—C7	106.9 (2)	N4—C16—H16A	109.00
C1—N2—C7	104.4 (2)	N4—C16—H16C	110.00
S1—N3—C14	115.8 (2)	N6—C17—C22	110.0 (3)
C14—N4—C16	121.4 (3)	N6—C17—C18	130.0 (3)
C14—N4—C15	121.6 (3)	C18—C17—C22	120.0 (3)
C15—N4—C16	117.0 (3)	C17—C18—C19	117.7 (4)
C6—N1—H1	127.00	C18—C19—C20	122.3 (3)
C7—N1—H1	127.00	C19—C20—C21	120.8 (3)
C22—N5—C23	107.1 (2)	C20—C21—C22	117.1 (3)
C17—N6—C23	104.5 (2)	N5—C22—C21	132.8 (3)
S2—N7—C30	115.9 (3)	N5—C22—C17	105.1 (2)
C30—N8—C32	119.6 (4)	C17—C22—C21	122.0 (3)
C31—N8—C32	118.2 (3)	N6—C23—C24	124.7 (3)
C30—N8—C31	122.3 (3)	N5—C23—C24	122.0 (3)
C23—N5—H5A	126.00	N5—C23—N6	113.2 (3)
C22—N5—H5A	126.00	C23—C24—C25	117.6 (3)
N2—C1—C6	110.0 (3)	C23—C24—C29	124.0 (3)
C2—C1—C6	119.4 (3)	C25—C24—C29	118.5 (3)
N2—C1—C2	130.6 (3)	C24—C25—C26	120.7 (3)
C1—C2—C3	118.6 (3)	C25—C26—C27	120.5 (3)
C2—C3—C4	121.3 (3)	C26—C27—C28	120.2 (3)
C3—C4—C5	121.3 (3)	C27—C28—C29	119.9 (3)
C4—C5—C6	116.9 (3)	S2—C29—C24	123.0 (2)
N1—C6—C5	132.5 (3)	S2—C29—C28	116.6 (2)
C1—C6—C5	122.3 (3)	C24—C29—C28	120.3 (3)
N1—C6—C1	105.1 (2)	N7—C30—N8	122.9 (4)
N1—C7—N2	113.6 (3)	C17—C18—H18	121.00
N2—C7—C8	125.2 (3)	C19—C18—H18	121.00
N1—C7—C8	121.1 (3)	C18—C19—H19	119.00
C9—C8—C13	118.3 (3)	C20—C19—H19	119.00
C7—C8—C13	124.6 (3)	C19—C20—H20	120.00
C7—C8—C9	117.1 (3)	C21—C20—H20	120.00
C8—C9—C10	120.7 (3)	C20—C21—H21	121.00
C9—C10—C11	120.5 (3)	C22—C21—H21	121.00
C10—C11—C12	119.9 (3)	C24—C25—H25	120.00
C11—C12—C13	120.2 (3)	C26—C25—H25	120.00
S1—C13—C12	116.2 (2)	C25—C26—H26	120.00
C8—C13—C12	120.4 (3)	C27—C26—H26	120.00
S1—C13—C8	123.3 (2)	C26—C27—H27	120.00
N3—C14—N4	123.6 (3)	C28—C27—H27	120.00
C3—C2—H2	121.00	C27—C28—H28	120.00
C1—C2—H2	121.00	C29—C28—H28	120.00
C2—C3—H3	119.00	N7—C30—H30	119.00
C4—C3—H3	119.00	N8—C30—H30	119.00
C3—C4—H4	119.00	N8—C31—H31A	109.00
C5—C4—H4	119.00	N8—C31—H31B	109.00
C4—C5—H5	122.00	N8—C31—H31C	109.00
C6—C5—H5	122.00	H31A—C31—H31B	110.00
C10—C9—H9	120.00	H31A—C31—H31C	109.00
C8—C9—H9	120.00	H31B—C31—H31C	109.00
C9—C10—H10	120.00	N8—C32—H32A	109.00
C11—C10—H10	120.00	N8—C32—H32B	109.00
C12—C11—H11	120.00	N8—C32—H32C	109.00
C10—C11—H11	120.00	H32A—C32—H32B	109.00
C11—C12—H12	120.00	H32A—C32—H32C	109.00
C13—C12—H12	120.00	H32B—C32—H32C	109.00
			
O1—S1—N3—C14	−2.2 (3)	C1—C2—C3—C4	0.4 (7)
O2—S1—N3—C14	129.0 (3)	C2—C3—C4—C5	−1.3 (8)
C13—S1—N3—C14	−118.6 (3)	C3—C4—C5—C6	0.4 (7)
O1—S1—C13—C8	−70.0 (3)	C4—C5—C6—N1	179.1 (4)
O1—S1—C13—C12	106.1 (3)	C4—C5—C6—C1	1.5 (6)
O2—S1—C13—C8	165.6 (3)	N2—C7—C8—C13	82.7 (4)
O2—S1—C13—C12	−18.3 (3)	N1—C7—C8—C9	77.7 (4)
N3—S1—C13—C8	50.6 (3)	N1—C7—C8—C13	−101.6 (4)
N3—S1—C13—C12	−133.3 (2)	N2—C7—C8—C9	−98.0 (4)
O4—S2—N7—C30	−127.7 (3)	C7—C8—C13—S1	−5.2 (4)
C29—S2—N7—C30	119.9 (3)	C7—C8—C13—C12	178.9 (3)
O3—S2—C29—C24	67.4 (3)	C13—C8—C9—C10	−0.7 (4)
O3—S2—C29—C28	−108.7 (3)	C7—C8—C9—C10	180.0 (3)
O4—S2—C29—C24	−168.1 (3)	C9—C8—C13—C12	−0.4 (4)
O4—S2—C29—C28	15.9 (3)	C9—C8—C13—S1	175.5 (2)
O3—S2—N7—C30	3.0 (3)	C8—C9—C10—C11	1.1 (5)
N7—S2—C29—C28	130.5 (2)	C9—C10—C11—C12	−0.5 (5)
N7—S2—C29—C24	−53.4 (3)	C10—C11—C12—C13	−0.6 (5)
C7—N1—C6—C5	−177.8 (4)	C11—C12—C13—C8	1.1 (5)
C6—N1—C7—N2	−0.1 (4)	C11—C12—C13—S1	−175.2 (2)
C7—N1—C6—C1	0.2 (4)	N6—C17—C18—C19	179.5 (4)
C6—N1—C7—C8	−176.2 (3)	C22—C17—C18—C19	−1.5 (6)
C7—N2—C1—C6	0.0 (4)	N6—C17—C22—N5	0.6 (4)
C7—N2—C1—C2	−179.5 (4)	N6—C17—C22—C21	−177.6 (4)
C1—N2—C7—N1	0.1 (4)	C18—C17—C22—N5	−178.6 (3)
C1—N2—C7—C8	176.0 (3)	C18—C17—C22—C21	3.3 (6)
S1—N3—C14—N4	−179.6 (3)	C17—C18—C19—C20	−1.0 (7)
C15—N4—C14—N3	−1.3 (6)	C18—C19—C20—C21	2.0 (8)
C16—N4—C14—N3	178.0 (4)	C19—C20—C21—C22	−0.3 (7)
C23—N5—C22—C21	177.1 (4)	C20—C21—C22—N5	−179.9 (4)
C22—N5—C23—N6	0.8 (4)	C20—C21—C22—C17	−2.3 (6)
C23—N5—C22—C17	−0.8 (4)	N5—C23—C24—C25	−81.3 (4)
C22—N5—C23—C24	176.6 (3)	N5—C23—C24—C29	99.8 (4)
C23—N6—C17—C22	−0.2 (4)	N6—C23—C24—C25	94.1 (4)
C17—N6—C23—N5	−0.4 (4)	N6—C23—C24—C29	−84.9 (4)
C23—N6—C17—C18	178.9 (4)	C23—C24—C25—C26	−178.3 (3)
C17—N6—C23—C24	−176.1 (3)	C29—C24—C25—C26	0.7 (5)
S2—N7—C30—N8	179.8 (3)	C23—C24—C29—S2	3.5 (4)
C31—N8—C30—N7	2.3 (6)	C23—C24—C29—C28	179.5 (3)
C32—N8—C30—N7	−177.3 (4)	C25—C24—C29—S2	−175.5 (2)
C6—C1—C2—C3	1.3 (6)	C25—C24—C29—C28	0.5 (4)
N2—C1—C6—N1	−0.1 (4)	C24—C25—C26—C27	−1.1 (5)
N2—C1—C2—C3	−179.2 (4)	C25—C26—C27—C28	0.3 (5)
N2—C1—C6—C5	178.1 (4)	C26—C27—C28—C29	0.9 (5)
C2—C1—C6—N1	179.5 (3)	C27—C28—C29—S2	174.9 (2)
C2—C1—C6—C5	−2.3 (6)	C27—C28—C29—C24	−1.3 (5)

### Hydrogen-bond geometry (Å, º)

**Table:**

*D*—H···*A*	*D*—H	H···*A*	*D*···*A*	*D*—H···*A*
N1—H1···N2^i^	0.86	2.11	2.964 (3)	174
N5—H5*A*···N6^ii^	0.86	2.10	2.955 (3)	178
C9—H9···O4^i^	0.93	2.56	3.153 (4)	122
C10—H10···O4^i^	0.93	2.57	3.153 (4)	121
C25—H25···O2^ii^	0.93	2.47	3.114 (5)	127

Symmetry codes: (i) −*x*+1/2, *y*−1/2, −*z*+1/2; (ii) −*x*+3/2, *y*+1/2, −*z*+1/2.
